# Is maternal defensiveness of Gyr cows *(Bos taurus indicus*) related to parity and cows’ behaviors during the peripartum period?

**DOI:** 10.1371/journal.pone.0274392

**Published:** 2022-09-09

**Authors:** Rogério Ribeiro Vicentini, Lenira El Faro, Aska Ujita, Maria Lúcia Pereira Lima, André Penido Oliveira, Aline Cristina Sant’Anna

**Affiliations:** 1 Núcleo de Estudos em Etologia e Bem-estar Animal (NEBEA), Universidade Federal de Juiz de Fora (UFJF), Juiz de Fora, Minas Gerais, Brasil; 2 Centro Avançado de Pesquisa de Bovinos de Corte, Instituto de Zootecnia (IZ)—Agência Paulista de Tecnologia dos Agronegócios/Secretaria de Agricultura e Abastecimento (APTA/SAA), Sertãozinho, São Paulo, Brazil; 3 Empresa de Pesquisa Agropecuária de Minas Gerais (EPAMIG Oeste), Uberaba, Minas Gerais, Brasil; 4 Departamento de Zoologia, Núcleo de Estudos em Etologia e Bem-estar Animal (NEBEA), Universidade Federal de Juiz de Fora (UFJF), Conselho Nacional de Desenvolvimento Científico e Tecnológico–CNPq Researcher, Juiz de Fora, Minas Gerais, Brazil; Universidade Federal de Mato Grosso do Sul, BRAZIL

## Abstract

The maternal care of cows can influence both the milk production and the performance of their calves, making this a topic of important relevance for the production industry that uses zebu cattle. The aims of this study were to 1) investigate the effects of parity on the behaviors of Gyr cows during the peripartum period; 2) characterize the maternal defensiveness of primiparous and multiparous cows towards handlers during the first handling of their calves; and 3) evaluate the relationships between cows’ behaviors at the peripartum period and maternal defensiveness. Thirty-one Gyr cows (primiparous and multiparous), from Empresa de Pesquisa Agropecuária de Minas Gerais (Brazil), were used. The animals were placed in a maternity paddock monitored by video cameras. The behaviors of the animals were collected in four periods: Pre-calving, Post-calving, First handling of calf and Post-handling. Primiparous cows presented more pain signs, reflected in arched spine (P = 0.05), and tended to move more (P = 0.07) than the multiparous in the Pre-calving period. Trends were observed for both Maternal Composite Score (P = 0.06) and Maternal Protective Behavior score (P = 0.06), indicating that both primiparous and multiparous were protective, but only multiparous cows were aggressive toward the caretakers on the first handling of their calves. The most protective cows spent more time eating during the prepartum period (P = 0.03), while the least attentive cows spent more time lying down (P = 0.02) in the prepartum period. The cows who nursed and stimulated their calves more were also calmer (P = 0.02) and more attentive (P = 0.01). In conclusion, the peripartum behaviors of Gyr cows were related to maternal care and maternal defensiveness. Multiparous cows tended to be more aggressive than primiparous cows at the time of the first handling of their calves.

## Introduction

Despite the process of domestication and intense artificial selection in dairy cattle, resulting in several breeds and behavioral changes in relation to their wild ancestors [1], the behaviors related to maternal care and protection of offspring have been maintained in some breeds [2]. These behaviors are important components and desirable for offspring survivorship and development in extensive cattle production systems [3–5]. After birth, a strong mother-offspring bond is formed [6]. The bond formation is mediated mainly by olfactory, visual, and auditory stimuli that result in reciprocal individual recognition [7]. Good quality cow-calf interactions soon after birth are important to assure better chances of offspring survival [5]. Experienced cows (*i*.*e*. multiparous) usually have a shorter latency to investigate and stimulate the calf compared to inexperienced cows (*i*.*e*. primiparous) [6, 8]. In addition to the parity, genetic and environmental factors might also affect the maternal behavior of cows [9, 10]. These behaviors, such as investigation, stimulation, maternal care, and defensiveness, can be used to characterize the maternal style, suggesting that these animals might have stable inter-individual differences that could be regarded as the maternal temperament [5, 11].

As a prey species, an important component of the maternal style is defensiveness, since the calf should be under the dam’s constant care in order to provide protection against predators and/or threatening conspecifics during the first weeks of life [12]. Although maternal defense behaviors are necessary and desirable under more natural conditions, in farming production systems, cows displaying extreme protection responses in relation to their calf might threaten or attack handlers during the first handling of their calves. Such extreme reactions can raise the risks of on-farm accidents, injury to handlers and animals, and even threaten the calves’ welfare, leading to physical damages or abandonment of calves [4, 13].

Previous studies have aimed to investigate the maternal care and the expression of maternal protective behaviors in cattle herds, mainly focusing on beef cattle breeds [3, 4, 9, 14–17], and only a few have been conducted with dairy cattle [18, 19]. This is a relevant question in cow-calf contact dairy systems. For Zebu cattle (*Bos taurus indicus*), the maternal defensiveness behavior was assessed in beef Gyr, Brahman, and their crossbreed cows [14, 17] and Holstein-Gyr crossbred dairy cows [19]. In the study of Pérez-Torres et al. [14], 90% of the cows displayed defensive behavior when the handler was close to the calf at 30 days after birth. These defensive reactions were strengthened when the calves vocalized or were handled. It is possible that the cows perceived humans as potential predators [14]. Evaluating multiparous cows, Orihuela et al. [17] reported that cows reacting more protectively to separation from their calves also exhibited more aggressive behaviors towards the handlers. However, the author failed to find a relationship between maternal protectiveness and the cows’ temperament in the peripartum period [17]. Ceballos et al. [19] reported that Holstein-Gyr crossbred cows who were more aggressive during the handling of their calves tended to be characterized as more ‘frightened’ and ‘active’ than those regarded as ‘loving’ and ‘attentive’ towards their calves when assessed using a qualitative behavior assessment, evincing a possible relationship between maternal defensiveness and the maternal style of care.

These issues are relevant in dairy herds of Zebu dairy herds in which the calves are not separated from their dams early post-birth, as is typical in European dairy breeds (*Bos taurus taurus*). The lack of stimuli from the calves compromises the length of the lactation period, that can be shortened, in Zebu cows, therefore the use of cow-calf contact systems is common for these animals [10, 20]. In dairy systems using Zebu breeds, the maternal defense and the mother-offspring bond are important, since the cows are known to not fully adapt to machine milking and being milked with their calves [21, 22].

Thus, the aims of the present study were to: 1) investigate the effects of parity on the behaviors of Gyr cows during the peripartum period; 2) characterize the maternal defensiveness of primiparous and multiparous cows towards the handlers during the first handling of their calves; and 3) evaluate the relationships between cows’ behaviors at the peripartum period and maternal defensiveness.

## Material and methods

The experiment was conducted at the Getúlio Vargas Experimental Station, Empresa de Pesquisa Agropecuária de Minas Gerais (EPAMIG), Uberaba, Minas Gerais State, Brazil (19º 44’ 54" S latitude and 47º 55’ 55" W longitude, altitude of 801 m) and was approved by the Ethics Committee of Animal Use of the Instituto de Zootecnia, Nova Odessa, São Paulo State, Brazil (CEUA/IZ 230–16).

### Animals and handling

Thirty-one Gyr cows (*Bos taurus indicus*), primiparous (n = 16) and multiparous (n = 15) were used. The animals were aged between 30 to 132 months and calved between July and December 2017. The calving order of multiparous cows ranged from two to six calvings. Thirty days before the estimated calving day, cows were transferred from the pasture to a maternity paddock of 0.55 ha size. The maximum stocking density in the maternity paddock was 27.27 animals/ha, and all cows had access to natural shade. The paddock was covered with *Urochloa decumbens* grass. Cows were fed with corn silage and 500 g of concentrate/head delivered twice a day, in addition to mineral supplements and water *ad libitum*. During the study period, cows were not handled or disturbed. Only routine procedures (feeding, calves’ identification and navel disinfection) were conducted by the usual familiar handlers.

### Behavioral observations

In the maternity paddock, cows were individually identified with non-toxic paint. Four monitoring cameras (GIGA, GSHDP20TB) were installed in the paddock corners to record the cows’ behaviors 24 hours a day.

In this study, only eutocic and non-twin calvings were included. The calving moment was defined as the complete expulsion of the fetus. After calving, a minimum period of 3 hours was permitted for cows and calves to remain together without any human disturbance. Afterward, the first handling for calf inspection and navel disinfection was conducted. The calves handling in the studied farm occurred daily from 8 am to 5 pm. Cows that delivered from 5 pm to 4 am remained with their calves longer (from 3h to 12h) undisturbed before the first handling of the calf since the farm handlers did not work overnight. The navel disinfection was conducted by two handlers familiar to the animals and previously trained in a standardized way: (1) the handlers remained still in the entrance of the paddock for 15 s enabling cows to have visual contact and be aware of their presence; (2) handlers walked towards the cow with an equable and non-threatening posture (lowered arms and avoiding eye contact with the cow), approaching laterally at an angle of 45° with the ventrodorsal cow axis; (3) one of the handlers roped the calf with a long rope and brought it closer to the fence while the other handler observed the cows for safety reasons; (4) both handlers crossed the paddock fence to exit the paddock; (5) the handlers drove the calf outside the paddock under the fence using the rope, inspected it and performed the navel disinfection using a commercial antiseptic (Umbicura^®^—Pecuarista d’Oeste), allowing the cows to have visual contact with her calf; (6) after the navel disinfection the handler removed the rope and drove the calf back to the paddock crossing under the fence.

For the behavioral recording, four periods were considered: (1) ‘Pre-calving Period’: 6 hours before calving (before the complete expulsion of the calf); (2) ‘Post-calving Period’: 3 hours after the complete expulsion of the calf; (3) ‘First handling’: the period of calf handling, including inspection and navel disinfection; (4) ‘Post-handling Period’: from the completion of navel disinfection to 1 hour later. A total of 560 hours of video recordings were analyzed (10 hours / animal). A single observer recorded the cows’ behaviors using focal sampling and continuous observation [23].

At ‘Pre-calving Period’, the behavioral categories ‘moving’, ‘feeding’, and ‘body posture’ were recorded, as described in Table 1, measured as the percentage of observation time (%). At ‘Post-calving’ and ‘Post-handling’ periods, behaviors related to the cow-calf interaction were recorded as described in Table 1, also measured as the percentage of observation time (%). The latencies for the cow to touch her calf (Cow latency), and for the calf to stand on their feet (Calf latency) were recorded in minutes. The following additional information regarding the calving was recorded: *i*) calving period (morning/afternoon/night), *ii*) delivery body posture (standing/ lying down), *iii*) distance of the calving cow from the herd (in meters), and *iv*) calf sex.

**Table 1:** Ethogram of Gyr cows behaviors and their calves in the peripartum period.

Categories	Description
*‘Pre-calving Period’*	
**Kneeling** (oc.^a^)	Cow’s forelegs bent on the floor and hindlegs erect.
**Drinking** (oc.)	Cow drinking water in the water bowl.
**Grazing** (%^a^)	Cow taking grass on the ground and showing chewing movement.
**Feeding** (%)	Cow eating silage and concentrates from the trough. Cow with the head above the trough and showing chewing movement.
**Straight spine** (%)	Cow standing with all four legs erects and spine straight.
**Arched spine** (%)	Cow standing with all four legs erect and arched spine.
**Moving** (%)	Cow walking forward or backward.
**Lying down** (%)	Lying in lateral or sternal decubitus, with the lower part of the body on the floor and legs stretched or retracted.
*‘Post-calving Period’* and *‘Post-handling Period’*	
**Cow latency** (min.^a^)	Period between the complete expulsion of the fetus (calving) until the cow touches the calf for the first time with muzzle and/or tongue.
**Calf latency** (min.)	Period between the expulsion of the fetus (calving) until the calf stands itself on four legs without falling.
**Touching** (%)	Cow’s tongue or muzzle keeping physical contact with any part of the calf’s body.
**Not interacting** (%)	Cow standing or lying without physical contact and/or without interacting with the calf.
**Suckling** (%)	Cow standing still while the calf sucks on her teats or makes contact with the teats and/or udder region.
**Moving** (%)	Cow walking forward or backward.

^a^oc. = occurrences (in number); % = percentage of observation time; min = latency in minutes.

At ‘First handling’ period, the cow protectiveness was assessed by a single trained observer using the video recordings. A ‘*Maternal Protection Scoring System’* was assigned, in which scores were attributed to ‘*Aggressiveness*’ (1 to 3), ‘A*ttention’* (1 to 3), ‘*Displacement*’ (1 to 5), and ‘*Agitation*’ (1 to 4) according to Ceballos et al. [19]. These scores were then added to compose a single scale the *Maternal Composite Score* (MCS). The sum of the scores for Aggressiveness, Attention, Displacement, and Agitation ranged from 4 (min.) to 11 (max.), generating a MCS from 1 to 8. In addition, a single grade for ‘*Maternal Protective Behavior*’ (MPB) was applied from 1 to 5, in which lower scores were indifferent and less protective cows and higher were more defensive and nervous cows (Table 2).

**Table 2:** Maternal Protective Behavior (MPB) score of Gyr cows at the first handling of their calves.

Scores	Descriptions
1	Calm cow; remains standing still.
2	Cow runs away from the handler and leaves the calf alone.
3	Cow shows signs of nervousness; flaps the tail; snorts; vocalizes.
4	Cow stands between the handler and calf with nervousness signs, not allowing the handlers to approach the calf.
5	Cow reacts aggressively, threatening^a^ and/or attacking^b^ the handler.

^a^Threatens: Stares at the handler with head up or head down; presents continuous head movement and/or displacement towards the handler, but does not attack;

^b^Attacks: vigorous displacement towards the handler, followed by physical contact with the fence (usually head-butts).

### Statistical analyses

Descriptive statistics and tests of normality were conducted for all behavioral variables using the PROC Univariate (SAS^®^ Institute, INC., Cary, NC). To evaluate the effect of parity on the cows’ behaviors, general linear models were fitted, using the PROC GLM (SAS^®^ Institute, INC., Cary, NC). The behaviors at pre-calving (Grazing, Feeding, Straight spine, Arched spine, Moving, Lying down); post-calving (Cows’ latency, Calves’ latency, Touching, Not interacting); and post-handling periods (Touching, Not interacting); in addition to MCS and MPB scores were used in the models as dependent variables. The fixed effects of parity (multiparous *vs*. primiparous) and age of the cow (in months) as a covariate with linear effect were included. For variables with non-normal distribution (‘Kneeling’, ‘Drinking’, ‘Suckling’, and ‘Moving’) the parities were compared using non-parametric statistics (Mann-Whitney test).

A principal component analysis (PCA) was used to investigate the structure of correlation among the behavioral variables at pre-calving, post-calving and post-handling. The behaviors at ‘Pre-calving’ (Kneeling, Drinking, Grazing, Feeding, Straight spine, Arched spine, Moving, Lying down), ‘Post-calving’ (Cows’ latency, Calves’ latency, Touching, Not interacting, Suckling, Moving), and ‘Post-handling’ periods (Touching, Not interacting, Suckling, Moving) were included in a matrix of animals (*rows*) per behaviors (*columns*). Principal components (PC) with eigenvalues above 1 were retained, and variables with loadings above 0.5 were regarded as the main contributors to the PC.

The Pearson’s correlation coefficient was used to investigate the relationships between the maternal protectiveness scores are the cows’ behaviors at the three periods and the PC obtained in the PCA. In all analyses, *P-*values ≤ 0.05 were regarded as significant, and P-values ≤ 0.10 were discussed as tendencies.

## Results

### Cows’ behaviors at pre-calving, post-calving, and post-handling periods

Regarding the calving time, 22.6% occurred in the morning, 45.2% in the afternoon, and 32.2% at night. Most of the cows delivered lying down (90.3%), and only 9.7% delivered standing up. Regarding the calving distance from the herd, 38.7% of the cows calved ‘very close’ (≤ 1 m) to the herd, 12.9% calved ‘close’ (> 1 and ≤ 4 m), 6.5% calved ‘next’ (> 4 and ≤ 6 m), and 41.9% calved ‘away’ (> 6 m) from the herd. For the calf sex, 51.6% were male and 48.4% female (S1 Table).

The most prevalent behavioral category at Pre-calving period was standing with ‘Straight spine’. Primiparous and multiparous cows differed for the behavior ‘Arched spine’ (F = 4.23; P = 0.05) and a tendency was found for ‘Moving’ (F = 3.58; P = 0.07). Primiparous cows had three times more standing with ‘Arched spine’ and tended to move more than the multiparous (Table 3).

**Table 3:** Means (± standard deviation) of Gyr cows behaviors in the peripartum period.

Cows’ behavior	Mean±Std	Primiparous	Multiparous
*‘Pre-calving Period’*			
**Kneeling** (oc.^a^)	1.29±2.81	1.23±3.58	1.35±1.98
**Drinking** (oc.)	1.40±1.33	1.30±1.25	1.50±1.45
**Grazing** (%^a^)	10.63±13.32	9.23±10.84	15.58±85.46
**Feeding** (%)	5.45±4.38	5.13±4.41	5.75±4.49
**Straight spine** (%)	36.97±16.07	32.93±16.63	40.17±15.16
**Arched spine** (%)	10.65±12.87	15.71±16.00^A^	5.95±6.77^B^
**Moving** (%)	13.57±8.46	16.70±10.16^A^	10.69±5.40^B^
**Lying down** (%)	22.74±13.93	20.34±13.87	24.96±14.11
*‘Post-calving Period’*			
**Cows’ latency** (min.^a^)	6.09±19.93	10.81±28.24	1.65±1.76
**Calves’ latency** (min.)	60.61±40.38	68.87±44.66	52.82±35.48
**Touching** (%)	50.76±16.51	49.39±16.80	52.03±16.72
**Not interacting** (%)	44.78±17.77	45.64±17.47	44.00±16.62
**Suckling** (%)	3.51±4.69	4.05±4.52	3.00±4.95
**Moving** (%)	0.95±2.01	0.93±2.30	0.97±1.78
*‘Post-handling Period’*			
**Touching** (%)	34.75±19.01	36.87±16.41	32.63±84.49
**Not interacting** (%)	59.73±19.44	56.46±17.10	62.99±21.58
**Suckling** (%)	3.44±5.35	3.96±5.86	2.92±4.92
**Moving** (%)	2.08±6.13	2.71±8.00	1.46±3.59

^a^oc. = number of occurrences; % = relative frequency; min = latency in minutes.

^A–B^ Different letters in the same line indicate significance (P ≤ 0.05) or tendency (P ≤ 0.10).

At the Post-calving period, the most prevalent category was ‘Touching’ the calf, and at the Post-handling period, it was ‘Not interacting’ with the calf (Table 3). Primiparous and multiparous cows did not differ (P > 0.05) in the Post-calving and Post-handling behaviors.

**Fig 1:**
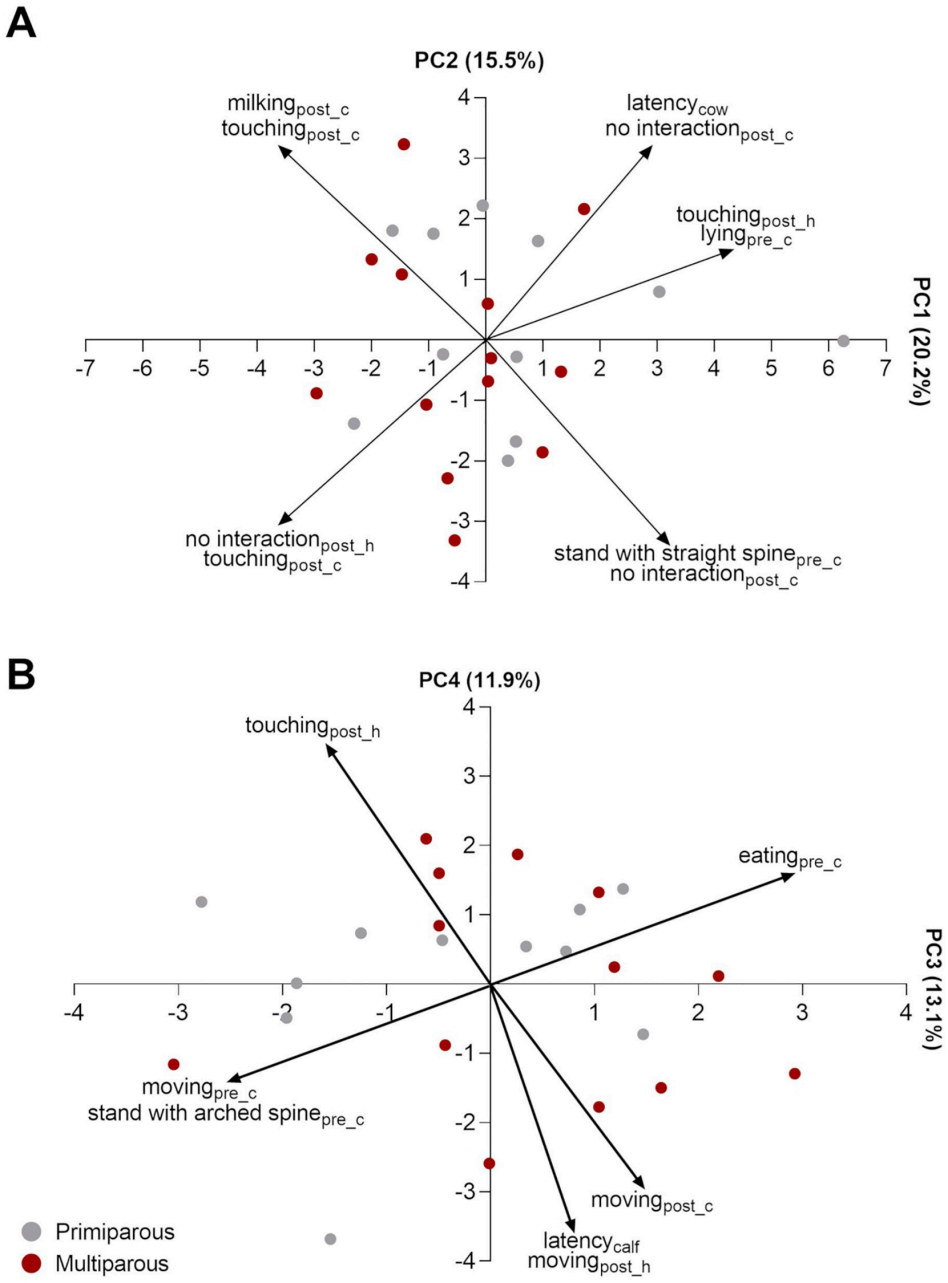
Plot of animals in the PC1 vs. PC2 (A) and PC3 vs. PC4 (B) extracted using behavioral data of Gyr primiparous (gray) and multiparous (red) cows at peripartum period (n = 24). Where pre_c = pre-calving, post_c = post-calving and post_h = post-handling periods.

In the PCA, four PC had eigenvalues above 1 and, together, explained 60.74% of the total variance in the dataset (Table 4). The PC1 explained 20.24% of the total variance and had higher loadings for ‘Lying down’ (Pre-calving), ‘Cows’ latency’, ‘Not interacting’ (Post-calving), and ‘Touching’ (Post-handling), while higher negative loadings were found for ‘Not interacting’ (Post-handling) and ‘Touching’ (Post-calving). This axis might have distinguished cows that spent more time lying down at Pre-calving period, spent more time without interacting with the calf, and took longer to interact with the calf (higher scores in PC1) from those who touched the calf more frequently at Post-calving and less frequently at Post-handling period (lower scores in PC1) (Fig 1A).

**Table 4:** Principal components analysis of Gyr cows and their calves’ behaviors in peripartum period.

Behaviors	PC1	PC2	PC3	PC4
Cows’ latency	**0.711** ^a^	-0.011	-0.140	0.097
Calves’ latency	0.453	0.133	0.241	**-0.674**
Straight spine _pre_c_^b^	-0.395	**-0.628**	-0.010	0.378
Feeding _pre_c_	0.216	-0.462	**0.563**	0.135
Moving _pre_c_	-0.190	-0.166	**-0.721**	0.010
Lying down _pre_c_	**0.727**	0.272	0.187	-0.179
Arched spine _pre_c_	0.287	0.305	**-0.657**	-0.128
Touching _post_c_^b^	**-0.577**	**0.617**	-0.114	0.152
Not interacting _post_c_	**0.574**	**-0.733**	0.070	-0.108
Suckling _post_c_	-0.157	**0.759**	0.235	0.096
Moving _post_c_	-0.368	-0.097	-0.405	**-0.564**
Touching _post-h_^b^	**0.561**	0.292	-0.256	**0.644**
Not interacting _post_h_	**-0.663**	-0.339	0.308	-0.330
Moving _post_h_	0.207	0.037	-0.334	**-0.708**
Eigenvalue	3.643	2.793	2.364	2.136
Variance explained (%)	20.24	15.51	13.13	11.86

^a^Values in bold represent the higher contributions to each PC (above 0.5);

^b^pre_c = pre-calving period; post_c = post-calving period; post_h = post-handling period.

The PC2 explained 15.51% of the total variance and had higher positive loadings for ‘Suckling’ (Post-calving) and ‘Touching’ (Post-calving), and negative for ‘Straight spine’ (Pre-calving) and ‘Not interacting’ (Post-calving) (Table 4). This axis ranged from cows that spent more time touching and suckling their calves at Post-calving period (higher scores in PC2), to those who spent more time standing with straight spine at Pre-calving and less time interacting with their calves at Post-calving period (lower scores in PC2), (Fig 1A). Therefore, cows’ scores in this PC can be an indicator of the frequency of nursing behavior at Post-calving period.

The PC3 explained 13.13% of the variance, showing higher positive loading for ‘Feeding’ (Pre-calving), and negative loading for standing with ‘Arched spine’ (Pre-calving) and ‘Moving’ (Pre-calving) (Table 4). This PC might have reflected the comfort/discomfort of cows at Pre-calving period, ranging from cows that spent more time eating (less evidence of discomfort) to those who spent more time moving and standing with arched spine (more evidence of discomfort) (Fig 1B).

Finally, PC4 (10.70% of variance) had higher positive loading for ‘Touching’ (Post-handling), and negative for ‘Cows’ latency’, ’Moving’ (Post-calving) and ’Moving’ (Post-handling). This axis ranged from cows that spent more time touching their calves at Post-handling period, to those who moved more at Post-calving and Post-handling and had longer latency to touch their calves (Fig 1B).

### Maternal defensiveness

**Fig 2:**
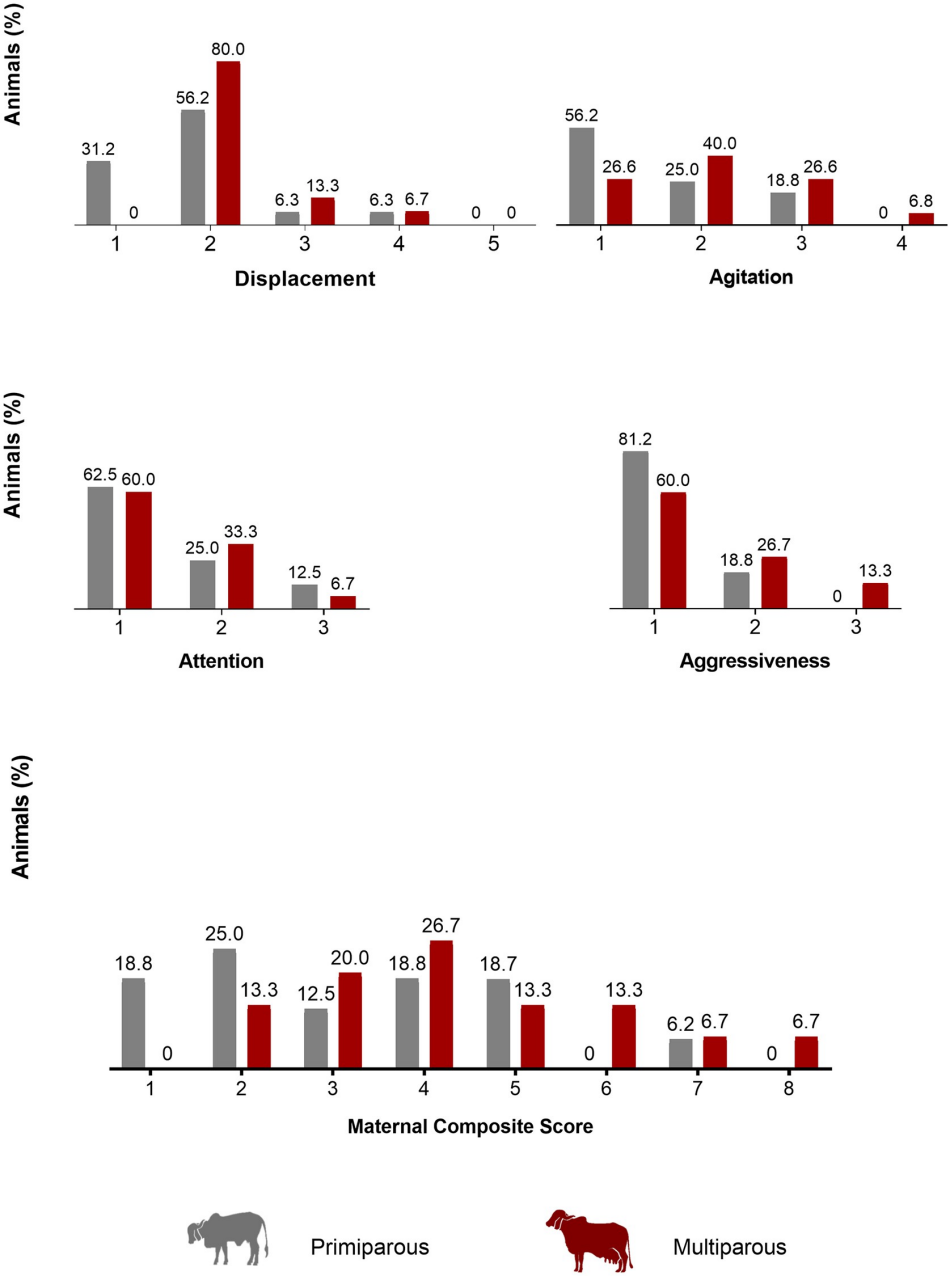
Maternal protection scoring system of primiparous and multiparous Gyr cows at the first handling of their calves (n = 31).

**Fig 3:**
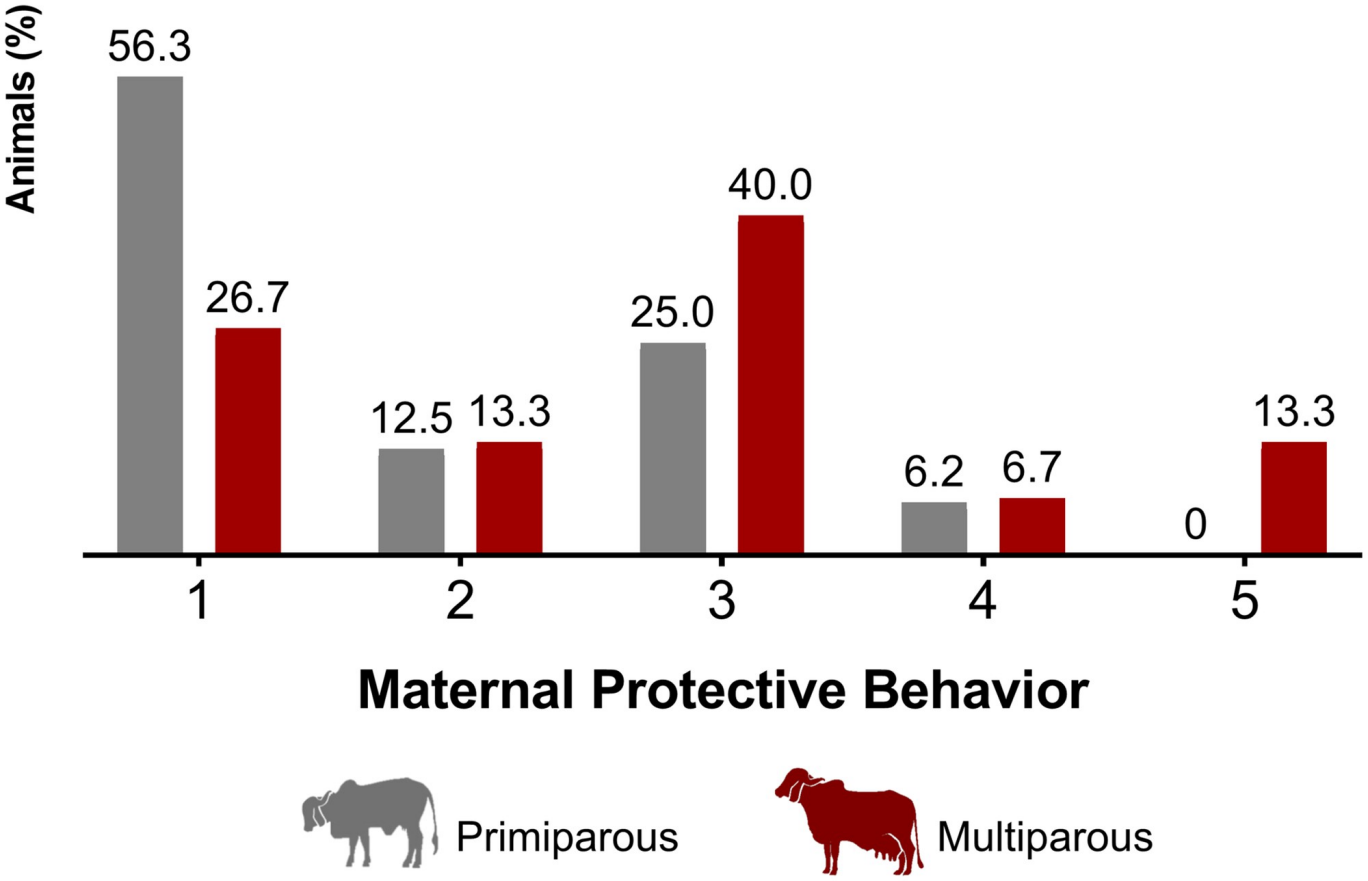
‘Maternal Protective Behavior’ (MPB) of primiparous and multiparous Gyr cows at the first handling of their calves (n = 31).

The distributions for the ‘Displacement’, ‘Agitation’, ‘Attention’ and ‘Aggressiveness’ scores and ‘*Maternal Composite Score’* (MCS) are displayed in Fig 2, and ‘*Maternal Protective Behavior*’ (MPB) distribution is shown in Fig 3. The parity showed a tendency on MCS (F = 3.57; P = 0.06) and MPB (F = 3.65; P = 0.06) scores. Multiparous cows had higher grades for both scores (4.40±1.76; 3.27±1.79, respectively) than the primiparous cows (3.19±1.76; 2.12±1.50, respectively), indicating that the multiparous tended to be more protective than primiparous cows.

### Relationships between cows’ behaviors at peripartum and maternal defensiveness

The maternal defensiveness scores were correlated with cows’ behavioral categories. A positive correlation was found between the MPB score and ‘Feeding’ behavior at Pre-calving (r = 0.425; P = 0.030), indicating that more protective cows spent more time eating in the feeder at Pre-calving period. A positive correlation was also found between ‘Displacement’ score and ‘Moving’ behavior Post-calving (r = 0.579; P = 0.008), showing that cows that displaced more during their calves handling also moved more in the Post-calving period. Finally, a negative correlation was found between ‘Attention’ score and ‘Lying down’ behavior (r = -0.444; P = 0.023). Cows characterized as less attentive to their calves’ handlings spent more time laying down during Pre-calving period.

The maternal defensiveness scores were also correlated with the four PC obtained in the PCA. A negative correlation was found between MPB and ‘PC2 (r = -0.457; P = 0.02), showing that cows that spent more time nursing (touching and suckling their calves, with higher scores in PC2), had lower MPB being calmer and less nervous/aggressive during the handling of their calves. In addition, ‘PC4’ was negatively correlated with ‘Displacement’ score (r = -0.529; P = 0.07) and positively with ‘Attention’ score (r = 0.495; P = 0.01). Cows that spent more time touching their calves and had lower latency to touch the calf (higher scores in PC4) tended to move less at Post-handling period and were characterized as more attentive during the first handling of their calves.

## Discussion

The maternal behavior of cows can influence both the milk production and the performance of their calves, making this a topic of important relevance for the production industry. In addition, issues related to animal welfare and caretakers’ safety can also be impacted by the management and behavior of cows in the peripartum period. The objectives of this study were to characterize the behavior of primiparous and multiparous Gyr cows in the peripartum period and its relationship with maternal defense during the first handling of their calves. Parity was related to both peripartum behavior and maternal protection. Primiparous cows showed more signs of pain and discomfort during the prepartum period than multiparous cows. Both primiparous and multiparous were protective, but only multiparous cows were aggressive ones. The peripartum behavior and maternal protectiveness were also related. The most protective cows ate for the most time in the prepartum period, while the least attentive were the ones that spent more time lying down in the prepartum period. The cows that nursed, stimulated, and touched their calves more frequently were also calmer and more attentive.

The higher incidence of daytime calvings (morning and afternoon) may be related to the selective advantages of calving at different times of the day, or may even result from conditioning the animals to the farm routine [6]. Among the factors that can influence the period of calving, Proudfoot et al. [24] highlighted changes in light patterns, diurnal hormones, and management routine. Regarding calving position, the vast majority of cows calved lying down, corroborating what is already described in the literature as the most frequent calving position by cows [25]. The position of calving must be taken into account as an important practical factor and indicator of difficulties in the calving process. Albeit zebu cattle show a lower frequency of dystocia, the cows’ posture during parturition may indicate obstetric problems, from which there is a greater risk of calf death when the cow gives birth standing up [26, 27]. Regarding the calving distance, cows usually tend to move away from the herd in the early hours before calving and looking for a quieter and hidden place. This distancing behavior has an adaptive value that may be preserved in domestic species, avoiding the risk of offspring death by predators and other threats [2]. However, we emphasize that this behavior, whether moving away from the herd (separation behavior) or not (aggregation behavior), is a phenotype with plasticity potential influenced by several factors [3, 24, 28]. In cattle and other domestic ungulate species, calving females can only distance themselves from the herd when the environment is favorable (*e*.*g*., presence of shelter, dense and natural vegetation, topography condition); otherwise, they calve in the herd [2, 24, 29]. In our study, the maternity paddock had no shelter or natural vegetation, which may have led the cows to calve closer to each other. The size of the maternity paddock (smaller than pasture areas) and proximity to management facilities (with a high frequency of traffic of working machinery and people) may also be related to a higher incidence of calving cows close to the herd in this study.

At the final gestation period, both fetal growth increase and energetic mobilization by the fetus can influence the behavior of cows, promoting the reduction of feed intake and movements. In the hours before calving, cows become more restless [6, 30]. The higher frequencies of feeding behavior and resting behavior compared to other behaviors may be related to physiological changes prior to the calving. Cows tend to decrease their food and water consumption before giving birth, but not completely. In the study by Jensen (31), Holstein cows decreased but did not stop water and food intake in the hours before calving. The rupture of the amniotic sac seems to be responsible for stimulating consumption since it relieves pressure in the abdominal region of cows [31, 32]. In relation to moving behavior, previous studies both in European cattle [31, 33–36] and Zebu cattle [37], demonstrated that in the hours before calving cows tend to move more. Huzzey et al. [33] observed an increase of standing bouts of Holstein cows housed in free-stall systems during the calving day using pedometer devices. Using behavioral observation by video recordings, Miedema et al. [30] and Jensen [31] described increased frequency of lying bouts of Holstein Frisian cows kept indoors six hours before calving. In Holstein cows kept on pasture, Rice et al. [38] found an increase of lying bouts between three and four hours before calving through pedometers. In a similar field condition to the present study, using intra-ruminal transponders, a previous study with Gyr cows showed an increase of activity 11 hours before calving [37].

The increased activity and movements can be related to pain and discomfort, signs from myometrial contractions, and the fetus expulsion [6, 39]. In our study, primiparous cows moved more and spent more time with an arched spine, which we understand as signs that show more effort and discomfort in the parturition process than multiparous cows. Cattle are known to arch their spines under physiological and pathological situations. During the delivery process [39], vaginal exams [40], and in severe cases of laminitis [41], the arched spine seems to be directly related to pain and discomfort. There are also anatomical and physiological differences between age, and consequently, parity (*e*.*g*., organ size, shape, cervical dilation) which contributes to facilitating the calving process for multiparous cows, who showed fewer discomfort signs in the present study [25, 31].

The first component of PCA (PC1) revealed variables related to the maternal investigation. After calving, the cow’s attention tends to be directed towards the newborn immediately, and a strong cow-calf bond is established [6, 7]. Cows that take longer to touch their calves can compromise the quality of this bond. Cows with higher scores in PC1 spent more time lying down in the Pre-calving period and stayed longer time with no interaction, taking longer to touch their calves for the first time. We could infer that the relationship between cows that spent more time lying down in the Prepartum period with the greatest latency in touching their calves may be due to exhaustion from the labor process. Edwards and Broom (25) observed an association between cow exhaustion and delay in standing up soon after calving. Other factors, like environmental (*e*.*g*., presence of predators, weather conditions) and physiological (*e*.*g*., calf weight, calves’ vigor) conditions, can influence the time of calf investigation and stimulation by the dam [26, 42, 43]. In the second PC (PC2), variables with higher loadings were those related to maternal nursing and stimulation. Good quality of stimulation and maternal care in the early hours of life ensure the survival and good performance of the calves [43]. In PC2, cows that touch their calves more frequently also suckle the calf longer and sooner after calving, showing higher scores in PC2. In addition to maternal care, the success of the first suckling is crucial for offspring survival [44]. Schmidek et al. [45] state that the calf’s first suckling should occur within the early 3 hours of life. Therefore, cows that suckled the calf earlier and longer can be considered as having better maternal performance.

In turn, PC3 reflected variables related to comfort/discomfort of cows in the Pre-calving period, ranging from cows that spent more time feeding at the Pre-calving period to those who spent more time moving with arched spine. As previously discussed, the movement and arched spine posture might indicate pain and distress in the calving process. Mainau and Manteca [39] attribute these pain signs to physiological alterations caused by calving, the widening of the cervix’s and accentuated myometrial contractions. On the other hand, the feed intake behavior may be related to the absence of severe pain or relief of abdominal pressure by rupture of the amniotic sac [32].

The variables in PC4 also reflected the maternal nursing behaviors, ranging from cows that spent more time touching their calves after handling to those who spent more time moving and showed higher latency to touch their calves after birth. The cows can perceive their calves’ first handling as a potential threat [14]. After the reunion of cow and calf after handling, it is natural for the cow to lick and smell the calf, investigating it. Both cows [46], goats [47], and ewes [48] are known to lick their offspring after a separation period. Animals that moved more during the post-calving and post-handling periods may have tried to distance themselves from other cows and handlers in an attempt to protect the calf. Cows that are more frightened and perceive threats around them spend more time in vigilance, and this can result in negative effects on the latencies to stand up and first suckling, taking longer time to touch their calves after birth [26, 44].

The investigation, stimulation, and nursing are components (traits) of maternal behavior that play an important role in the calves’ health and safety. Similarly, maternal protection also assists in better chances of offspring survival [12]. Regarding maternal defense, our results showed that both multiparous and primiparous Gyr cows tend to move less and be more attentive during the handling of their calves. However, multiparous cows tended to have higher scores for agitation and aggressiveness than primiparous ones. These results suggest two main styles for the dams who were characterized as defensive in Gyr cows: ’Protective-attentive mothers’ and ’Protective-aggressive mothers’. Along these lines, in our study those cows defined as ‘Protective-attentive mothers’ were the cows that were alert and attentive during calves’ handling but did not threaten or attach the handlers. In turn, ‘Protective-aggressive mothers’ were those cows who were attentive and hostile (threatening and/or attacking) during their calves’ handling. The expression and intensity of maternal defense may reflect several individual factors such as temperament, body condition, and sex and vigor of offspring, in addition to previous experience and parity that seem to influence this behavior [11, 25, 43, 49, 50]. In taurine cattle, previous studies also showed that multiparous cows were more protective than primiparous ones when their calves were handled [9, 13, 18].

To our knowledge, there have been no previous reports evaluating maternal defensive behavior related to parity in zebu cattle; however, some studies have been conducted evaluating other aspects of maternal defensiveness in this subspecies. According to Pérez-Torres et al. [14], Gyr and Brahmans cows showed higher intensity of maternal protection until 90 days postpartum. For these animals, offspring protection seems to be so important that cows defended both their own and other cows’ calves [14]. In studies with Gyr and Brahman cows, Orihuela et al. [17] found no relationship between temperament and maternal defense in the peripartum period. However, cows more reactive to calves’ handling were those with more aggressive behavior toward humans [17]. Ceballos et al. [19] investigated maternal protectiveness in Holstein/Gyr crossed cows, reporting that the aggressive cows were also more frightened, irritated, and agitated during handling. Zebu cattle are widely known to be more reactive to handling than European cattle [22, 51, 52], and all these findings may suggest that more excitable behavior can also be seen in terms of maternal defense in some cows. Our results also indicate that the exacerbated defensiveness of multiparous Gyr cows observed in this study might suggest that even animals habituated to handling routines can react strongly to the handling of their newborn calves [5, 9]. The newborn care practices are essential for their health (*e*.*g*. navel asepsis, antiparasitic medicine, suckling assistance) but require close contact between the calving cows and the handlers [5, 43]. So, the aggressive cows may be a severe one-welfare problem, increasing the risk of stress and labor accidents for both handlers and animals.

The correlations showed a relationship between pre-calving and post-calving behaviors with maternal defense. The most defensive cows were the ones that spent more time feeding in the pre-calving period. Stěhulová et al. [50] reported that cows in better body conditions are more protective with their calves, demonstrating a relationship between feed-intake and maternal defense. Furthermore, these animals with greater feed-intake were also those showing fewer signs of pain and discomfort during the calving process, suggesting that cows without signs of severe pain or less weariness are those that defend their calves more. Edwards and Broom (25) reported the influence of exhaustion on delayed standing after calving, which can, in part, explain the relationship found between weariness and defensive behavior in Gyr cows in this study.

A correlation was also found between ‘Displacement’ score during the calves’ handling and the time spending moving after calves’ handling. This behavior may reflect the cows’ disturbance caused by their calves’ handling. Some cows may perceive the caretakers as a threat, and this displacement behavior during the handling can result from a nervous emotional state [14, 19]. We attributed the moving behavior after handling as an evasive strategy to move away with their calves from this perceived threat. The ‘Attention’ score was correlated with ‘Lying’ behavior during pre-calving, so cows less attentive to the handling of their calves spent more time lying down in the pre-calving period. The lying down position is related to the final stages of calving however can also be related to weariness, as discussed above [25]. Therefore, the less attentive cows may have been wearier due to the strain of the calving process.

The MPB was negatively correlated to PC2, indicating that higher scores in PC2 (cows spending more time nursing and touching their calves) had lower defensiveness scores (calmer and non-aggressive during the calves’ handling). Maternal protectiveness is positive and beneficial in the herds in which calves and cows are kept together. It is important that the dam licks and stimulates the offspring, facilitating recognition and contributing to a strong bond [53–55]. Likewise, it is also important that cows do not attack caretakers. In the practical context, desirable cows in the herd are those with good maternal ability that nurture and protect the calf, and accept their handling.

Similarly, the correlation between MPB and PC4 showed that cows with better maternal nursing behaviors moved less and were more attentive to the handling of their calves. These results indicate that cows with better maternal performance (lower latency in touching the calf after calving, suckling longer, and touched their calves more) also had better maternal temperament. Thus, we could infer that cows labeled as ’Protective-attentive mothers’ and presenting a lower risk of danger to the handlers were better mothers than ’Protective-aggressive mothers’ cows. Our results indicated that both multiparous and primiparous cows were protective, but only multiparous cows were regarded as aggressive. While these findings suggest that primiparous cows did not present any aggressive behavior towards caretakers, it is not clear if it was a result of weariness from the calving process (more intense in primiparous) or if perhaps there was some stimulus during the calf management process that had triggered the aggressive behavior in multiparous cows. Our results also bring a new perspective on maternal defense behavior in zebu cattle, highlighting the implications of cow behavior in the peripartum period. Further studies to better understand maternal aggressiveness and the factors that influence it may enhance the management efficiency in dairy farms and cow-calf operations, ensuring the safety of handlers and caretakers.

## Conclusions

In conclusion, the peripartum behaviors of both primiparous and multiparous Gyr cows are related to the dam interactions with the calf and maternal defensiveness. Primiparous cows showed more behavioral signs of pain and discomfort during prepartum, which may have affected their interaction with their calves. Multiparous cows showed less behavior indicative of pain and discomfort during the parturition process. Both primiparous and multiparous cows tended to be protective, but only multiparous cows showed aggressive behavior towards the caretakers. The most protective cows spent more time feeding, while less attentive cows spent more time lying down during the pre-calving period. Cows with better maternal performance (nursing, stimulation and touching their calves) were calmer, moved less, and were more attentive during the handling of their calves.

## Supporting information

S1 Table. Relative frequency (%) of calving period, calving position, calving distance, and calves’ sex of Gyr cows by parity (DOCX)

S1 Data (XLSX)

## References

[1] Decker JE, McKay SD, Rolf MM, Kim J, Molina Alcalá A, Sonstegard TS, et al. Worldwide Patterns of Ancestry, Divergence, and Admixture in Domesticated Cattle. PLOS Genetics. 2014;10(3):e1004254. doi: 10.1371/journal.pgen.1004254 24675901 PMC3967955

[2] Rørvang MV, Nielsen BL, Herskin MS, Jensen MB. Prepartum Maternal Behavior of Domesticated Cattle: A Comparison with Managed, Feral, and Wild Ungulates. Frontiers in Veterinary Science. 2018;5(45). doi: 10.3389/fvets.2018.00045 29594159 PMC5857534

[3] Flörcke C, Engle TE, Grandin T, Deesing MJ. Individual differences in calf defence patterns in Red Angus beef cows. Applied Animal Behaviour Science. 2012;139(3):203–8. doi: 10.1016/j.applanim.2012.04.001

[4] Turner SP, Jack MC, Lawrence AB. Precalving temperament and maternal defensiveness are independent traits but precalving fear may impact calf growth1. Journal of Animal Science. 2013;91(9):4417–25. doi: 10.2527/jas.2012-5707 23825324

[5] Costa FdO, Valente TdS, Paranhos da Costa MJR, del Campo M. Expressão do comportamento de proteção materna em bovinos: uma revisão. Revista Acadêmica Ciência Animal. 2018;16. doi: 10.7213/1981-4178.2018.161106

[6] von Keyserlingk MAG, Weary DM. Maternal behavior in cattle. Hormones and Behavior. 2007;52(1):106–13. doi: 10.1016/j.yhbeh.2007.03.015 17490663

[7] Kent JP. The cow–calf relationship: from maternal responsiveness to the maternal bond and the possibilities for fostering. Journal of Dairy Research. 2020;87(S1):101–7. Epub 2020/07/29. doi: 10.1017/S0022029920000436 33213588

[8] Kiley-Worthington M, de la Plain S. Fostering and Adoption in Beef Cattle. The Behaviour of Beef Suckler Cattle (Bos Taurus). Basel: Birkhäuser Basel; 1983. p. 144–57.

[9] Hoppe S, Brandt HR, Erhardt G, Gauly M. Maternal protective behaviour of German Angus and Simmental beef cattle after parturition and its relation to production traits. Applied Animal Behaviour Science. 2008;114(3):297–306. doi: 10.1016/j.applanim.2008.04.008

[10] Upadhyay VKT A.K.S; Patel B.H.M; Golher D.M.; Sahu S.; Bharti P.K. Effect of early weaning on milking behaviour, production and reproduction of Tharparkar cows. Indian Journal of Dairy Science. 2015;68(5).

[11] Fairbanks LA. Individual Differences in Maternal Style: Causes and Consequences for Mothers and offspring. In: Rosenblatt JS, Snowdon CT, editors. Advances in the Study of Behavior. 25: Academic Press; 1996. p. 579–611.

[12] Lent PC. Mother-infant relationships in ungulates. The behaviour of ungulates and its relation to management. 1974;1:14–55.

[13] Buddenberg BJ, Brown CJ, Johnson ZB, Honea RS. Maternal Behavior of Beef Cows at Parturition. Journal of Animal Science. 1986;62(1):42–6. doi: 10.2527/jas1986.62142x 3957810

[14] Pérez-Torres L, Orihuela A, Corro M, Rubio I, Cohen A, Galina CS. Maternal protective behavior of zebu type cattle (Bos indicus) and its association with temperament1. Journal of Animal Science. 2014;92(10):4694–700. doi: 10.2527/jas.2013-7394 25149346

[15] Vallée A, Breider I, van Arendonk JAM, Bovenhuis H. Genetic parameters for large-scale behavior traits and type traits in Charolais beef cows1. Journal of Animal Science. 2015;93(9):4277–84. doi: 10.2527/jas.2015-9292 26440327

[16] Kohari D, Takakura A. Questionnaire investigation to clarify the occurrence rate and characteristics of maternal rejection behavior in Japanese black cattle (Bos taurus). Animal Science Journal. 2017;88(12):2071–6. doi: 10.1111/asj.12858 28799182

[17] Orihuela A, Pérez-Torres L, Ungerfeld R. The time relative to parturition does not affect the behavioral or aggressive reactions in Zebu cows (Bos indicus). Livestock Science. 2020;234:103978. doi: 10.1016/j.livsci.2020.103978

[18] Geburt K, Friedrich M, Piechotta M, Gauly M, König von Borstel U. Validity of physiological biomarkers for maternal behavior in cows—A comparison of beef and dairy cattle. Physiology & Behavior. 2015;139:361–8. doi: 10.1016/j.physbeh.2014.10.030 25446230

[19] Ceballos MC, Góis KCR, Sant’Anna AC, Wemelsfelder F, Paranhos da Costa M. Reliability of qualitative behavior assessment (QBA) versus methods with predefined behavioral categories to evaluate maternal protective behavior in dairy cows. Applied Animal Behaviour Science. 2021;236:105263. doi: 10.1016/j.applanim.2021.105263

[20] Orihuela A. Effect of calf stimulus on the milk yield of Zebu-type cattle. Applied Animal Behaviour Science. 1990;26(1):187–90. doi: 10.1016/0168-1591(90)90098-X

[21] Assan N. Influence of suckling and/or milking method on yield and milk composition in dairy animals. Scientific Journal of Zoology. 2015;4(1):1–7.

[22] Ujita A, El Faro L, Vicentini RR, Pereira Lima ML, de Oliveira Fernandes L, Oliveira AP, et al. Effect of positive tactile stimulation and prepartum milking routine training on behavior, cortisol and oxytocin in milking, milk composition, and milk yield in Gyr cows in early lactation. Applied Animal Behaviour Science. 2021;234:105205. doi: 10.1016/j.applanim.2020.105205

[23] Martin MBP. Measuring Behaviour: An Introductory Guide. 4th ed. Cambridge Cambridge University Press; 2021. 200 p.

[24] Proudfoot KL, Weary DM, von Keyserlingk MAG. Maternal isolation behavior of Holstein dairy cows kept indoors1. Journal of Animal Science. 2014;92(1):277–81. doi: 10.2527/jas.2013-6648 24371006

[25] Edwards SA, Broom DM. Behavioural interactions of dairy cows with their newborn calves and the effects of parity. Animal Behaviour. 1982;30(2):525–35. doi: 10.1016/S0003-3472(82)80065-1

[26] Paranhos da Costa M, Schmidek A, Toledo L. Mother–Offspring Interactions in Zebu Cattle. Reproduction in Domestic Animals. 2008;43(s2):213–6. doi: 10.1111/j.1439-0531.2008.01164.x 18638126

[27] Segura-Correa JC, Magaña-Monforte JG, Aké-López JR, Segura-Correa VM, Hinojosa-Cuellar JA, Osorio-Arce MM. Breed and environmental effects on birth weight, weaning weight and calving interval of Zebu cattle in southeastern Mexico. Tropical and Subtropical Agroecosystems. 2017;20(2):297–305.

[28] Lidfors LM, Moran D, Jung J, Jensen P, Castren H. Behaviour at calving and choice of calving place in cattle kept in different environments. Applied Animal Behaviour Science. 1994;42(1):11–28. doi: 10.1016/0168-1591(94)90003-5

[29] Alexander G, Stevens D, Bradley LR, Barwick SA. Maternal behaviour in Border Leicester, Glen Vale (Border Leicester derived) and Merino sheep. Australian Journal of Experimental Agriculture. 1990;30(1):27–38.

[30] Miedema HM, Cockram MS, Dwyer CM, Macrae AI. Changes in the behaviour of dairy cows during the 24h before normal calving compared with behaviour during late pregnancy. Applied Animal Behaviour Science. 2011;131(1):8–14. doi: 10.1016/j.applanim.2011.01.012

[31] Jensen MB. Behaviour around the time of calving in dairy cows. Applied Animal Behaviour Science. 2012;139(3):195–202. doi: 10.1016/j.applanim.2012.04.002

[32] Wehrend A, Hofmann E, Failing K, Bostedt H. Behaviour during the first stage of labour in cattle: Influence of parity and dystocia. Applied Animal Behaviour Science. 2006;100(3):164–70. doi: 10.1016/j.applanim.2005.11.008

[33] Huzzey JM, von Keyserlingk MAG, Weary DM. Changes in Feeding, Drinking, and Standing Behavior of Dairy Cows During the Transition Period. Journal of Dairy Science. 2005;88(7):2454–61. doi: 10.3168/jds.S0022-0302(05)72923-4 15956308

[34] Clark CEF, Lyons NA, Millapan L, Talukder S, Cronin GM, Kerrisk KL, et al. Rumination and activity levels as predictors of calving for dairy cows. Animal. 2015;9(4):691–5. doi: 10.1017/S1751731114003127 25491656

[35] Borchers MR, Chang YM, Proudfoot KL, Wadsworth BA, Stone AE, Bewley JM. Machine-learning-based calving prediction from activity, lying, and ruminating behaviors in dairy cattle. Journal of Dairy Science. 2017;100(7):5664–74. doi: 10.3168/jds.2016-11526 28501398

[36] Rutten CJ, Kamphuis C, Hogeveen H, Huijps K, Nielen M, Steeneveld W. Sensor data on cow activity, rumination, and ear temperature improve prediction of the start of calving in dairy cows. Computers and Electronics in Agriculture. 2017;132:108–18. doi: 10.1016/j.compag.2016.11.009

[37] Vicentini RR, Bernardes PA, Ujita A, Oliveira AP, Lima MLP, El Faro L, et al. Predictive potential of activity and reticulo-rumen temperature variation for calving in Gyr heifers (Bos taurus indicus). Journal of Thermal Biology. 2021;95:102793. doi: 10.1016/j.jtherbio.2020.102793 33454034

[38] Rice CA, Eberhart NL, Krawczel PD. Prepartum Lying Behavior of Holstein Dairy Cows Housed on Pasture through Parturition. Animals. 2017;7(4):32. doi: 10.3390/ani7040032 28420107 PMC5406677

[39] Mainau E, Manteca X. Pain and discomfort caused by parturition in cows and sows. Applied Animal Behaviour Science. 2011;135(3):241–51. doi: 10.1016/j.applanim.2011.10.020

[40] Pilz M, Fischer-Tenhagen C, Thiele G, Tinge H, Lotz F, Heuwieser W. Behavioural reactions before and during vaginal examination in dairy cows. Applied Animal Behaviour Science. 2012;138(1):18–27. doi: 10.1016/j.applanim.2012.01.011

[41] Flower FC, Weary DM. Effect of Hoof Pathologies on Subjective Assessments of Dairy Cow Gait. Journal of Dairy Science. 2006;89(1):139–46. doi: 10.3168/jds.S0022-0302(06)72077-X 16357276

[42] Toledo LM, Paranhos da Costa MJR, Titto EAL, Figueiredo LdA, Ablas DdS. Impactos de variáveis climáticas na agilidade de bezerros Nelore neonatos. Ciência Rura. 2007;37. doi: 10.1590/S0103-84782007000500028

[43] Pires BV, Freitas LAd, Silva GVd, Mendonça GG, Savegnago RP, Lima MLPd, et al. Maternal-offspring behavior of Guzerat beef cattle. Pesquisa Agropecuária Brasileira. 2020;55. doi: 10.1590/S1678-3921.pab2020.v55.01504

[44] Toledo LM, Paranhos da Costa MJR, Schmidek A, Jung J, Ciryllo JNSG, Cromberg VU. The presence of black vultures at the calving sites and its effects on cows’ and calves’ behaviour immediately following parturition. Animal. 2013;7(3):469–75. doi: 10.1017/S1751731112001735 23031159

[45] Schmidek A, Mercadante MEZ, Paranhos da Costa MJR, Razook AG, Figueiredo LAd. Falha na primeira mamada em bezerros Guzerá: fatores predisponentes e parâmetros genéticos. Revista Brasileira de Zootecnia. 2008;37(6):998–1004.

[46] le Neindre P, D’Hour P. Effects of a postpartum separation on maternal responses in primiparous and multiparous cows. Animal Behaviour. 1989;37(1):166–7. doi: 10.1016/0003-3472(89)90023-7

[47] Lickliter RE. Effects of a post-partum separation on maternal responsiveness in primiparous and multiparous domestic goats. Applied Animal Ethology. 1982;8(6):537–42. doi: 10.1016/0304-3762(82)90217-6

[48] Brown DJ, Fogarty NM, Iker CL, Ferguson DM, Blache D, Gaunt GM. Genetic evaluation of maternal behaviour and temperament in Australian sheep. Animal Production Science. 2016;56(4):767–74. doi: 10.1071/AN14945

[49] Pitts AD, Weary DM, Fraser D, Pajor EA, Kramer DL. Alternative housing for sows and litters.: Part 5. Individual differences in the maternal behaviour of sows. Applied Animal Behaviour Science. 2002;76(4):291–306. doi: 10.1016/S0168-1591(02)00012-6

[50] Stěhulová I, Špinka M, Šárová R, Máchová L, Kněz R, Firla P. Maternal behaviour in beef cows is individually consistent and sensitive to cow body condition, calf sex and weight. Applied Animal Behaviour Science. 2013;144(3):89–97. doi: 10.1016/j.applanim.2013.01.003

[51] Sant’Anna AC, Paranhos da Costa MJR, Baldi F, Rueda PM, Albuquerque LG. Genetic associations between flight speed and growth traits in Nellore cattle1. Journal of Animal Science. 2012;90(10):3427–32. doi: 10.2527/jas.2011-5044 22585807

[52] Lima MLP, Negrão JA, de Paz CCP, Grandin T. Minor corral changes and adoption of good handling practices can improve the behavior and reduce cortisol release in Nellore cows. Tropical Animal Health and Production. 2018;50(3):525–30. doi: 10.1007/s11250-017-1463-9 29139068

[53] Poindron P, Neindre PL. Endocrine and Sensory Regulation of Maternal Behavior in the Ewe. In: Rosenblatt JS, Hinde RA, Beer C, Busnel M-C, editors. Advances in the Study of Behavior. 11: Academic Press; 1980. p. 75–119.

[54] la Torre MPd, Briefer EF, Ochocki BM, McElligott AG, Reader T. Mother–offspring recognition via contact calls in cattle, Bos taurus. Animal Behaviour. 2016;114:147–54. doi: 10.1016/j.anbehav.2016.02.004

[55] Toledo LMd Fernandes TB, Paranhos da Costa MJR, Ambrósio LA. Modelling the Dynamics of Cow-Calf Dyadic Behavior. International Journal of System Dynamics Applications (IJSDA). 2018;7(4):1–19. doi: 10.4018/IJSDA.2018100101

