# Supplementary material for: Is maternal defensiveness of Gyr cows (Bos taurus indicus) related to parity and cows’ behaviors during the peripartum period?
Source: PLoS One. 2022 Sep 9;17(9):e0274392. doi: 10.1371/journal.pone.0274392 (PMC9462786; doi:10.1371/journal.pone.0274392)
Supplement: S1 Table. Relative frequency (%) of calving period, calving position, calving distance, and calves’ sex of Gyr cows by parity — (DOCX) [file pone.0274392.s001.docx]

| **Supplementary Table 1. Relative frequency (%) of calving period, calving position, calving distance, and calves’ sex of Gyr cows by parity.** | | | |
| --- | --- | --- | --- |
|  | **Total** | **Primiparous** | **Multiparous** |
| ***Calving period*** |  | | |
| Morning _(06:00 a.m. to 11:59 a.m.)_ | 22.6% (7/31) | 18.7% (3/16) | 26.7% (4/15) |
| Afternoon _(12:00 p. m. to 05:59 p.m.)_ | 45.2% (14/31) | 62.6% (10/16) | 26.7% (4/15) |
| Night _(06:00 p.m. to 05:59 a.m.)_ | 32.2% (10/31) | 18.7% (3/16) | 46.6% (7/15) |
| ***Calving positon*** |  |  |  |
| Lying down | 90.3% (28/31) | 93.7% (15/16) | 86.7% (13/15) |
| Standing up | 9.7% (3/31) | 6.3% (1/16) | 13.3% (2/15) |
| ***Calving distance*** |  |  |  |
| Very close _(1 ≤ m)_ | 38.7% (12/31) | 37.5% (6/16) | 40.0% (6/15) |
| Close _(> 1 and ≤ 4 m)_ | 12.9% (4/31) | 12.5% (2/16) | 13.3% (2/15) |
| Next _(> 4 and ≤ 6 m)_ | 6.5% (2/31) | 6.2% (1/16) | 6.7% (1/15) |
| Away _(> 6 m)_ | 41.9% (13/31) | 43.8% (7/16) | 40.0% (6/15) |
| ***Calves’ sex*** |  |  |  |
| Male _(♂)_ | 51.6% (16/31) | 62.5% (10/16) | 40.0% (6/15) |
| Female _(♀)_ | 48.4% (15/31) | 37.5% (6/16) | 60.0% (9/15) |
